# The effect of night shift work on daytime sleepiness and physiological health among pediatric nurses in Northern Ghana: a cross-sectional survey

**DOI:** 10.1038/s41598-026-52977-8

**Published:** 2026-05-11

**Authors:** Ruth Nimota Nukpezah, Moses Tadri, Rockson Adenyo Evans, Thomas Nalignakiya Niyagnan, Mudasir Mohammed Ibrahim

**Affiliations:** 1 School of Nursing and Midwifery, University for Development Studies, Tamale, Ghana; 2 Department of Internal Medicine (M3), Tamale Teaching Hospital, Tamale, Ghana

**Keywords:** Night shift work, Daytime sleepiness, Pediatric nurses, Physiological health, Ghana, Health care, Medical research, Physiology, Risk factors

## Abstract

Pediatric nurses constitute a critical workforce in Ghana due to the continuous and high-intensity demands of child care. However, their occupational health outcomes remain underexplored, particularly in northern Ghana. This study assessed the effect of night shift work on daytime sleepiness and physiological health among pediatric nurses in the Tamale Metropolis, Northern Ghana. A cross-sectional survey was conducted among 175 pediatric nurses. A stratified sampling technique was used to ensure proportional representation of pediatric nurses from each health facility, followed by simple random sampling for participant selection. Data were collected using a structured questionnaire incorporating the Epworth Sleepiness Scale (ESS) and Night Shift Physiological Health Assessment Scale (NSPHAS). Appropriate inferential statistics were used, with statistical significance set at *p* < 0.05. The mean daytime sleepiness score following night shift work was 10.12 (SD = 5.55), with 20.6% of participants exhibiting severe excessive daytime sleepiness. The highest chance of dozing was reported when lying down to rest in the afternoon (31.4%). Sleep disturbances recorded the highest mean physiological health score (2.87 ± 0.98), followed by gastrointestinal disturbances and eating habit disruptions (2.29 ± 0.91), and cardiovascular and physical strain (2.28 ± 0.91). Significant differences in physiological health outcomes were also observed across sociodemographic and work-related characteristics (*p* < 0.05). Night shift work is associated with increased daytime sleepiness and physiological health disturbances among pediatric nurses. Context specific interventions, including improved shift scheduling and targeted sleep health education, may help reduce these adverse outcomes.

**Supplementary Information:**

The online version contains supplementary material available at 10.1038/s41598-026-52977-8.

## Introduction

Globally, nearly one-fifth of the workforce is engaged in shift work, with healthcare systems relying heavily on night shift scheduling to ensure uninterrupted, 24-hour patient care delivery^1,2^. While this structure is essential for maintaining continuity of services, a growing body of evidence indicates that night shift work is associated with significant adverse health outcomes. These effects are primarily attributed to disruptions in circadian rhythm regulation and the normal homeostatic sleep–wake cycle^3^.

One of the most immediate and well-documented consequences of night shift work is excessive daytime sleepiness. Daytime sleepiness refers to an increased tendency to fall asleep or difficulty maintaining alertness during usual waking hours, often resulting from inadequate sleep duration or poor sleep quality^4^. It is widely recognized as a key indicator of impaired sleep–wake regulation and is frequently used in occupational health research as a marker of fatigue-related functional impairment^5,6^. Among healthcare workers, excessive daytime sleepiness is particularly concerning due to its implications for attention, cognitive performance, clinical decision-making, and overall patient safety^7^.

Night shift work alters cortisol and melatonin secretion patterns, thereby directly impairing sleep quality^8,9^. Empirical evidence consistently shows that nurses working night shifts report significantly poorer sleep quality and higher levels of daytime sleepiness compared with their day-shift counterparts, with insufficient recovery sleep between shifts further exacerbating these effects^8,10,11^. Extended working hours and heavy workloads contribute to sleep disturbances among night-shift nurses^12^.

Beyond sleep-related outcomes, night shift work has been associated with a wide range of adverse physiological health effects. These include musculoskeletal disorders, occupational stress, depression, anxiety, metabolic dysregulation, and weight gain, often driven by irregular eating patterns, circadian misalignment, and chronic fatigue^9,13–15^. More concerningly, evidence suggests a significant association between night shift work and cardiovascular disease, with studies reporting an estimated 8–20% increased risk of coronary events and cardiovascular mortality among night-shift workers. These conditions include hypertension, coronary artery disease, myocardial infarction, and ischemic stroke, all of which are linked to long-term circadian disruption and sustained physiological stress^16,17^.

In Ghana, night shift work remains an indispensable component of hospital service delivery, particularly in ensuring continuity of care in public health facilities^18^. Nurses frequently engage in night duty schedules to maintain 24-hour patient care and emergency responsiveness^18^. However, despite the known health implications associated with night shift work, evidence on its specific effects among nurses in Ghana remains limited.

Furthermore, pediatric nurses constitute a critical workforce due to the continuous and high-intensity demands of child healthcare delivery. They operate in highly sensitive clinical environments where patient conditions can change rapidly, requiring sustained attention, emotional resilience, and high levels of clinical vigilance. Studies have shown that pediatric nurses face considerable occupational challenges, including heavy workload demands and emotional strain associated with caring for critically ill children^19,20^. However, the specific health effects of night shift work within this subgroup remain underexplored, as they are often subsumed under the broader category of nursing staff in existing studies, thereby limiting a focused understanding of their unique occupational health risks. This generalization of nurses as a homogeneous group limits the understanding of context-specific occupational risks, particularly for pediatric nurses whose work environment, patient population, and care demands differ significantly from those in adult care settings. Consequently, the extent to which night shift work affects their physiological health and daytime functioning remains poorly understood in the Ghanaian context, especially in northern Ghana where empirical evidence is limited. In response to this gap, the present study adopted an empirical approach to assess the effect of night shift work on daytime sleepiness and physiological health among pediatric nurses in the Tamale Metropolis, Northern Ghana. The findings are expected to provide context-specific evidence to inform occupational health policies and improve workforce well-being within pediatric nursing practice.

## Materials and methods

### Study design

This study employed a cross-sectional design to assess the effect of night shift work on daytime sleepiness and physiological health among pediatric nurses in the Tamale Metropolis, Northern Ghana.

### Study setting

The study was conducted in three public health facilities within the Tamale Metropolis of the Northern Region of Ghana: Tamale Teaching Hospital (TTH), Northern Regional Hospital (NRH), and the Seventh Day Adventist (SDA) Hospital. TTH is a tertiary referral hospital and one of the largest teaching hospitals in Ghana, serving as a major referral center for the northern regions. NRH is a primary government hospital providing general and specialist services within the Tamale Metropolis. SDA Hospital is a private, faith-based primary healthcare facility offering both general and specialist care. In 2025, the pediatric nursing staff strength across the facilities was 200 at TTH, 31 at NRH, and 25 at SDA Hospital, giving a total population of 256 pediatric nurses across the three facilities.

### Study population

The study population comprised nurses working in pediatric units in the three selected health facilities within the Tamale Metropolis who were engaged in rotating night shift schedules. Nurses who worked exclusively day shifts, as well as those who were unavailable during the study period, were excluded from the study.

## Sample size and sampling techniques

The sample size for this study was determined using the Yamane^21^ sample size formula for a known population: $n=\frac{N}{{1+N{{\left( e \right)}^2}}}$, where *n* represents the sample size, *N* is the study population (256 pediatric nurses), and *e* is the margin of error set at 5%. Applying the formula,

$$n=\frac{{256}}{{1+256{{\left( {0.05} \right)}^2}}}{\mathrm{~}}=\frac{{256}}{{1.64}}{\mathrm{~}}={\mathrm{~}}157$$

Thus, the minimum required sample size was 157. To account for potential non-response, a 10% adjustment was applied, resulting in a final sample size of 175 participants. A stratified sampling technique was employed to ensure proportional representation of pediatric nurses from each health facility. Nurses were first stratified by their respective health facility, after which proportionate allocation was applied using the formula: *(Sample Size / Population Size × Stratum Size)*. Accordingly, 137 participants were selected from TTH (200/256 × 175), 21 from NRH (31/256 × 175), and 17 from SDA Hospital (25/256 × 175). Within each stratum, simple random sampling was used to select participants, ensuring that every eligible pediatric nurse had an equal chance of inclusion. This process involved obtaining the pediatric nurses’ shift schedule, which contained their names and ranks at each facility. The list was imported into statistical software (as described in the data analysis section), and the software’s random sampling function was used to generate the required number of participants. The selected individuals were then traced to their respective wards and invited to participate in the study.

### Data collection instrument

Data were collected using a structured questionnaire comprising three (3) sections. Section A captured participants’ sociodemographic and work-related characteristics, including age, gender, marital status, highest level of nursing education, number of children, religion, years of work experience, professional rank, and other relevant variables. Section B assessed daytime sleepiness following night shift work using the Epworth Sleepiness Scale (ESS) developed by Johns^22^. The ESS is a validated, self-administered instrument consisting of eight (8) items (e.g., “Sitting and reading”, “Watching television (TV)”) that measure the chance of dozing in common everyday situations. Participants who reported current or previous engagement in night shift work were instructed to respond based on their usual level of daytime sleepiness after night shift work. Each item was rated on a 4-point Likert scale ranging from 0 (would never doze) to 3 (high chance of dozing). The total ESS score was computed and interpreted as follows (with higher scores indicating greater levels of daytime sleepiness): lower normal daytime sleepiness (0–5), higher normal daytime sleepiness (6–10), mild excessive daytime sleepiness (11–12), moderate excessive daytime sleepiness (13–15), and severe excessive daytime sleepiness (16–24)^22,23^.

Section C assessed the effect of night shift work on physiological health using the Night Shift Physiological Health Assessment Scale (NSPHAS) developed by the researchers based on a review of relevant literature^24–26^. The scale comprises three (3) subscales: (1) Sleep Disturbances (8 items; e.g., “I experience poor-quality sleep after night shifts”), (2) Gastrointestinal Disturbances and Eating Habit Disruptions (7 items; e.g., “I experience indigestion or acid reflux after night shifts”), and (3) Cardiovascular and Physical Strain (7 items; e.g., “I feel physically weak or shaky after working a night shift”). Participants rated their responses on a 5-point Likert scale ranging from 1 (never) to 5 (always). Subscale scores were computed by averaging item responses, and the subscale with the highest mean score was considered to represent the predominant physiological health domain affected by night shift work among participants. For interpretation, mean scores were categorized as follows: 1.00–1.80 (very low symptom presence), 1.81–2.60 (low symptom presence), 2.61–3.40 (moderate symptom presence), 3.41–4.20 (high symptom presence), and 4.21–5.00 (very high symptom presence). Higher mean scores indicated greater severity of the respective physiological health domain associated with night shift work (Supplementary File 1).

### Validity and reliability

The validity and reliability of the study instrument were initially assessed through a pilot study conducted among 40 nurses at Tamale West Hospital (TWH) prior to the main data collection. The pilot testing identified minor issues related to item wording and repetition within the physiological health section, which were subsequently revised to enhance clarity and readability. No major concerns were identified regarding the overall clarity, relevance, or structure of the instrument. Further validity and reliability analyses were conducted using data from the main study sample. The Epworth Sleepiness Scale (ESS) demonstrated good internal consistency (Cronbach’s alpha = 0.85). The self-developed Night Shift Physiological Health Assessment Scale (NSPHAS) showed good convergent validity and excellent composite reliability, with high internal consistency across subscales: sleep disturbances (α = 0.93), gastrointestinal disturbances and eating habit disruptions (α = 0.89), and cardiovascular and physical strain (α = 0.90), as well as an overall Cronbach’s alpha of 0.95 (Supplementary File 2).

### Data collection procedure

Following ethical approval from the relevant institutional authorities, the researchers visited each selected health facility on pre-scheduled dates to commence data collection. Eligible participants were identified using staff lists obtained from the nursing administration in each facility to ensure that all pediatric nurses had an equal opportunity for inclusion. From these lists, participants were selected and invited to participate in the study. Prior to data collection, the purpose, significance, and voluntary nature of the study were clearly explained to each participant. Both verbal and written informed consent were obtained. Participation was entirely voluntary, and participants were assured that refusal to participate would not affect their professional standing or employment status. The questionnaires were then self-administered to participants within the pediatric wards. Participants were allowed to complete the questionnaires at their convenience to minimize disruption to clinical duties. Completed questionnaires were reviewed on-site for completeness, and where responses were missing, participants were politely asked to review and complete the omitted items.

### Data analysis

Data were analyzed using SAS JMP Professional Statistical Software (version 17.1). Descriptive statistics, including frequencies, percentages, means, and standard deviations, were computed to summarize participants’ characteristics and study variables. Prior to inferential analysis, the assumptions for parametric tests were assessed and satisfied. Multivariate linear regression analysis was conducted to identify factors associated with daytime sleepiness while adjusting for potential confounders. Multicollinearity was assessed using variance inflation factors (VIF), with results indicating no evidence of problematic multicollinearity (mean VIF = 2.74; range: 1.14–5.80, all < 10) (Supplementary File 3). Differences in physiological outcomes namely sleep disturbances, gastrointestinal disturbances and eating habit disruptions, and cardiovascular and physical strain were examined using one-way ANOVA and independent two-sample t-tests, as appropriate. Where significant overall differences were identified in the one-way ANOVA, post hoc comparisons were conducted using Tukey’s Honestly Significant Difference (HSD) test to determine the specific group differences contributing to the observed effects. No missing data were observed in the dataset; therefore, all analyses were conducted using complete case analysis. Statistical significance was set at *p* < 0.05, and all analyses were performed at a 95% confidence level.

### Ethical consideration

Ethical approval for the study was obtained from the University for Development Studies, Tamale (Approval No. UDS/RB/291/25). Additional administrative permission was secured from the selected public health facilities prior to data collection. Following these approvals, both verbal and written informed consent were obtained from all participants after a clear explanation of the study’s purpose, procedures, and significance. Participation was entirely voluntary, and participants were informed of their right to decline or withdraw from the study at any time without any consequences to their professional standing. Confidentiality and anonymity were strictly maintained throughout the study. This was ensured by excluding all personal identifiers from the questionnaires and assigning unique codes in place of names. Data were handled securely and used solely for research purposes. All study procedures were conducted in accordance with relevant ethical guidelines and regulations.

## Results

### Sociodemographic and work-related characteristics

A total of 175 nurses participated in the study, representing a response rate of 100%. Table 1 presents the sociodemographic and work-related characteristics of the participants. The majority of participants were aged 30 years or younger (53.1%), male (68.6%), and married (58.3%). Most participants held a bachelor’s degree in nursing (52.0%) and reported having one child (59.4%). Over half had less than five years of work experience (56.6%) and were predominantly staff nurses (32.0%). A higher proportion of participants indicated that they liked working night shifts (67.4%). Regarding exposure to night shift work, 21.7% had worked 1–5 weeks of night shifts in the past year, while 20.0% reported more than 20 weeks; however, 31.4% could not recall their duration. Additionally, 37.1% reported holding a second job. Notably, nearly half of the participants reported obtaining less than 4 h of sleep after a night shift (49.1%), indicating a high level of sleep deprivation.

**Table 1:** Sociodemographic and work-related characteristics of participants (*N* = 175).

Variable	Frequency	Percentage
Age in years		
≤ 30	93	53.1
> 30	82	46.9
Gender		
Female	55	31.4
Male	120	68.6
Marital status		
Married	102	58.3
Single	72	41.1
Separated	1	0.6
Highest level of nursing education		
Diploma	79	45.1
Bachelor’s degree	91	52.0
Master’s degree	5	2.9
Number of children		
1	104	59.4
2	41	23.4
3	17	9.7
> 3	13	7.4
Religion		
Islam	68	38.9
Christianity	104	59.4
African Traditional	3	1.7
Work experience		
< 5 years	99	56.6
5–10 years	65	37.1
> 10 years	11	6.3
Rank		
Staff nurse	56	32.0
Senior staff nurse	55	31.4
Nursing officer	44	25.1
Senior nursing officer	15	8.6
Principal nursing officer	5	2.9
Likes night shift work		
Yes	118	67.4
No	57	32.6
Total number of weeks worked on night shifts in the past year		
1–5 weeks	38	21.7
6–10 weeks	28	16.0
11–20 weeks	19	10.9
> 20 weeks	35	20.0
I cannot remember	55	31.4
Currently hold a second job (in addition to this nursing role)		
Yes	65	37.1
No	110	62.9
Number of hours of sleep gotten after a night shift work		
< 4 h	86	49.1
4–6 h	71	40.6
> 6 h	18	10.3

### Descriptive statistics of the main study variables

The descriptive statistics of the main study variables are presented in Table 2. The mean daytime sleepiness score among participants was 10.12 (SD = 5.55). With respect to physiological health outcomes, the mean subscale scores indicate that participants experienced moderate sleep disturbances (2.87 ± 0.98). In contrast, gastrointestinal disturbances and eating habit disruptions (2.29 ± 0.91), as well as cardiovascular and physical strain (2.28 ± 0.91), were generally at low levels.

**Table 2:** Descriptive statistics of the main study variables (*N* = 175).

Variable	Mean	SD	Mean score interpretation
Daytime sleepiness	10.12	5.55	-
Physiological health			
Sleep disturbances	2.87	0.98	Moderate sleep disturbances
Gastrointestinal disturbances and eating habit disruptions	2.29	0.91	Low gastrointestinal disturbances and eating habit disruptions
Cardiovascular and physical strain	2.28	0.91	Low cardiovascular and physical strain

Note: Interpretation for daytime sleepiness mean score was not provided, as the standard instrument reports categorical classification based on total scores.

### Daytime sleepiness

**Fig. 1:**
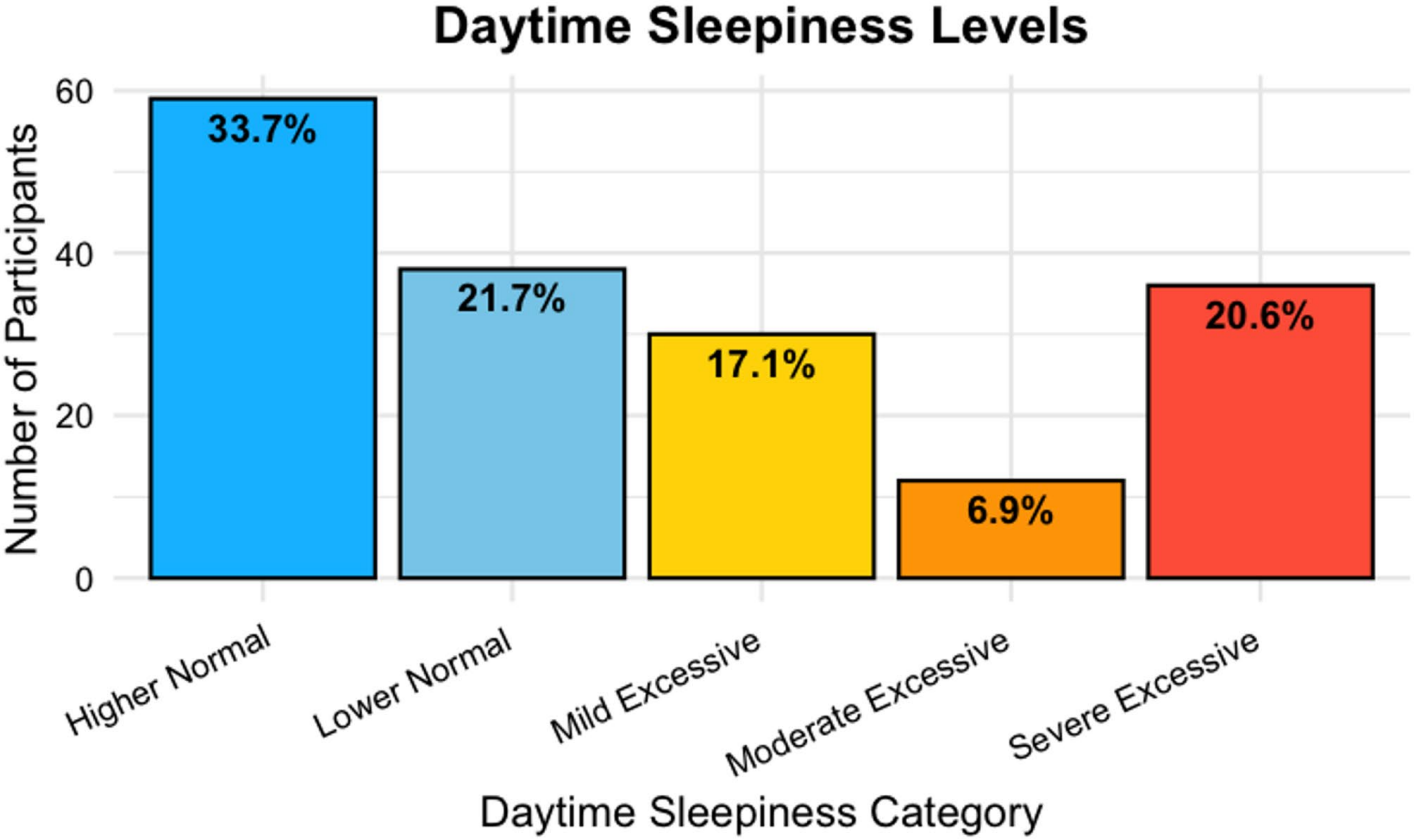
Daytime sleepiness levels among participants (*N* = 175).

Table 3; Fig. 1 present the distribution of daytime sleepiness among participants following night shift work. As illustrated in Fig. 1, 20.6% of participants experienced severe excessive daytime sleepiness (95% CI: 0.15–0.27). At the item level (Table 3), the highest chance of dozing was reported when participants were lying down in the afternoon to rest (31.4%). Moderate tendencies to doze were also commonly observed while watching television (35.4%) and when sitting quietly after lunch (32.6%). These findings indicate an increased propensity for sleep during passive or low-activity situations.

**Table 3:** Daytime sleepiness among participants (*N* = 175).

Variable	Would never doze (0)		Slight chance of dozing (1)		Moderate chance of dozing (2)		High chance of dozing (3)	
	*n*	%	*n*	%	*n*	%	*n*	%
1. Sitting and reading	47	26.9	67	38.3	35	20.0	26	14.9
2. Watching television (TV)	35	20.0	52	29.7	62	35.4	26	14.9
3. Sitting inactive in a public place	59	33.7	46	26.3	44	25.1	26	14.9
4. Sitting for an hour as a passenger in a car	31	17.7	49	28.0	53	30.3	42	24.0
5. Lying down in the afternoon to rest	23	13.1	44	25.1	53	30.3	55	31.4
6. Sitting and talking to another person	104	59.4	51	29.1	14	8.0	6	3.4
7. Sitting quietly after a lunch (no alcohol at lunch)	53	30.3	33	18.9	57	32.6	32	18.3
8. Sitting in a car, stopped for a few minutes due to traffic	75	42.9	60	34.3	26	14.9	14	8.0

### Physiological health

#### Sleep disturbances

Table 4 presents findings on sleep disturbances among participants following night shift work. At the item level, the highest mean score was recorded for “I sleep fewer hours after working at night” (3.16 ± 1.17), followed by “I find it hard to maintain a consistent sleep schedule while working nights” (3.05 ± 1.28) and “I experience poor-quality sleep after night shifts” (3.03 ± 1.22). The lowest mean score was observed for “I struggle to stay asleep during the day after a night shift” (2.60 ± 1.25).

**Table 4:** Sleep disturbances among participants (*N* = 175).

Variable	Never		Rarely		Sometimes		Often		Always		Mean (SD)
	*n*	%	*n*	%	*n*	%	*n*	%	*n*	%	
1. I sleep fewer hours after working at night	16	9.1	29	16.6	71	40.6	28	16.0	31	17.7	3.16 (1.17)
2. I find it hard to maintain a consistent sleep schedule while working nights	22	12.6	42	24.0	47	26.9	32	18.3	32	18.3	3.05 (1.28)
3. I experience poor quality sleep after night shifts	20	11.4	38	21.7	62	35.4	26	14.9	29	16.6	3.03 (1.22)
4. I take longer to fall asleep on days after working night duty	23	13.1	29	16.6	78	44.6	22	12.6	23	13.1	2.96 (1.16)
5. I wake up feeling unrested after sleeping post night shift	34	19.4	40	22.9	62	35.4	21	12.0	18	10.3	2.71 (1.20)
6. I struggle to stay asleep during the day after a night shift	44	25.1	35	20.0	61	34.9	17	9.7	18	10.3	2.60 (1.25)
7. I experience frequent sleep interruptions after night shifts	30	17.1	46	26.3	59	33.7	26	14.9	14	8.0	2.70 (1.15)
8. I feel drowsy or fall asleep unintentionally during the day after night duty	30	17.1	43	24.6	64	36.6	21	12.0	17	9.7	2.73 (1.17)

#### Gastrointestinal disturbances and eating habit disruptions

Table 5 depicts findings on gastrointestinal disturbances and eating habit disruptions associated with night shift work. The results show that these disturbances were primarily characterized by behavioral changes in eating patterns rather than clinical symptoms. The highest mean score was reported for “I eat at inappropriate times (e.g., midnight snacking) during night shifts” (2.66 ± 1.19), followed by “I eat more junk food or sugary snacks during night shifts” (2.49 ± 1.24), “I have irregular meal times while working night shifts” (2.39 ± 1.26), and “I skip meals while on night duty” (2.27 ± 1.15). The lowest mean score was observed for “I experience indigestion or acid reflux after night shifts” (1.79 ± 0.96).

**Table 5:** Gastrointestinal disturbances and eating habit disruptions among participants (*N* = 175).

Variable	Never		Rarely		Sometimes		Often		Always		Mean (SD)
	*n*	%	*n*	%	*n*	%	*n*	%	*n*	%	
1. I experience indigestion or acid reflux after night shifts	87	49.7	50	28.6	27	15.4	9	5.1	2	1.1	1.79 (0.96)
2. I have irregular meal times while working night shifts	58	33.1	35	20.0	53	30.3	13	7.4	16	9.1	2.39 (1.26)
3. I eat at inappropriate times (e.g., midnight snacking) during night shifts	37	21.1	37	21.1	65	37.1	20	11.4	16	9.1	2.66 (1.19)
4. I skip meals while on night duty	59	33.7	41	23.4	52	29.7	15	8.6	8	4.6	2.27 (1.15)
5. I feel bloated or gassy after meals during night shifts	63	36.0	45	25.7	44	25.1	10	5.7	13	7.4	2.22 (1.21)
6. I eat more junk food or sugary snacks during night shifts	48	27.4	42	24.0	51	29.1	19	10.9	15	8.6	2.49 (1.24)
7. I drink more caffeinated beverages to stay awake at night	71	40.6	41	23.4	38	21.7	12	6.9	13	7.4	2.17 (1.24)

#### Cardiovascular and physical strain

Table 6 presents findings on cardiovascular and physical strain among participants. The highest mean score was reported for “I feel fatigued throughout the day after night duty” (2.63 ± 1.21), followed by “I feel physically weak or shaky after working a night shift” (2.52 ± 1.26) and “I find it difficult to physically recover between night shifts” (2.32 ± 1.12). The lowest mean score was recorded for “I feel an increased heart rate while working night duty” (1.97 ± 1.07).

**Table 6:** Cardiovascular and physical strain among participants (*N* = 175).

Variable	Never		Rarely		Sometimes		Often		Always		Mean (SD)
	*n*	%	*n*	%	*n*	%	*n*	%	*n*	%	
1. I feel an increased heart rate while working night duty	76	43.4	49	28.0	37	21.1	6	3.4	7	4.0	1.97 (1.07)
2. I experience dizziness or light headedness while working nights	50	28.6	55	31.4	50	28.6	13	7.4	7	4.0	2.27 (1.08)
3. I feel unusually cold or hot during night shifts	68	38.9	41	23.4	39	22.3	24	13.7	3	1.7	2.16 (1.14)
4. I feel physically weak or shaky after working a night shift	52	29.7	31	17.7	54	30.9	25	14.3	13	7.4	2.52 (1.26)
5. I notice increased blood pressure or palpitations during night shifts	73	41.7	42	24.0	41	23.4	8	4.6	11	6.3	2.10 (1.18)
6. I feel fatigued throughout the day after night duty	41	23.4	36	20.6	59	33.7	25	14.3	14	8.0	2.63 (1.21)
7. I find it difficult to physically recover between night shifts	54	30.9	41	23.4	56	32.0	18	10.3	6	3.4	2.32 (1.12)

### Factors associated with daytime sleepiness

Multivariate linear regression analysis was conducted to identify sociodemographic and work-related factors associated with daytime sleepiness (Table 7). Three variables were found to be significantly associated with daytime sleepiness: religion, preference for night shift work, and holding a second job. Daytime sleepiness was significantly lower among Muslim participants compared to Christians (β = -2.07, SE = 0.87, 95% CI [-3.81, -0.33], *p* = 0.019). Participants who did not prefer night shift work reported significantly higher levels of daytime sleepiness compared to those who preferred night shifts (β = 2.33, SE = 0.97, 95% CI [0.41, 4.25], *p* = 0.017). Additionally, participants who did not hold a second job reported higher daytime sleepiness compared to those who held a second job (β = 1.81, SE = 0.92, 95% CI [0.01, 3.63], *p* = 0.050). All other variables, including age, gender, marital status, highest level of nursing education, number of children, work experience, rank, and number of hours of sleep after night shifts, were not significantly associated with daytime sleepiness (*p* > 0.05).

**Table 7:** Multivariate linear regression analysis of factors associated with daytime sleepiness among participants (*N* = 175).

Variable	Estimate (β)	Std. error	Sig.	95% CI lower	95% CI upper
Age in years					
≤ 30	Reference				
> 30	0.63	1.08	0.555	-1.49	2.77
Gender					
Male	Reference				
Female	-1.30	1.03	0.214	-3.34	0.75
Marital status					
Single	Reference				
Married	0.64	1.07	0.546	-1.47	2.76
Highest level of nursing education					
Diploma	Reference				
Bachelor’s degree	-0.53	1.11	0.634	-2.73	1.68
Master’s degree	3.47	3.36	0.303	-3.16	10.11
Number of children					
0	Reference				
1	0.13	1.78	0.939	-3.39	3.66
2	0.01	1.90	0.994	-3.76	3.78
3	-1.90	2.18	0.383	-6.22	2.41
Religion					
Christianity	Reference				
African Traditional	2.47	4.07	0.544	-5.56	10.51
Islam	-2.07	0.87	0.019*	-3.81	-0.33
Work experience					
> 10 years	Reference				
5–10 years	0.10	2.38	0.964	-4.59	4.81
< 5 years	-1.37	2.61	0.601	-6.54	3.80
Rank					
Staff nurse	Reference				
Senior staff nurse	-0.38	1.11	0.730	-2.58	1.81
Nursing officer	-1.75	1.50	0.244	-4.72	1.21
Senior nursing officer	0.89	2.10	0.669	-3.26	5.06
Principal nursing officer	-3.28	3.73	0.381	-10.66	4.10
Likes night shift work					
Yes	Reference				
No	2.33	0.97	0.017*	0.41	4.25
Currently hold a second job (in addition to this nursing role)					
Yes	Reference				
No	1.81	0.92	0.050*	0.01	3.63
Number of hours of sleep gotten after a night shift work					
v> 6 h	Reference				
4–6 h	1.72	1.48	0.248	-1.21	4.66
< 4 h	1.74	1.49	0.242	-1.19	4.69

*Statistically significant at *p* < 0.05.

### Differences in physiological health

Differences in physiological health subscale scores across key variables are summarized in Tables 8 and 9, and 10. As shown in Table 8, for the sleep disturbances domain, significant differences were observed based on religion, rank, and preference for night shift work. Participants practicing Christianity reported higher mean sleep disturbance scores (3.03 ± 0.96) compared with those practicing African Traditional religion (1.58 ± 0.71) (*p* = 0.028). Senior staff nurses reported higher mean scores (3.24 ± 0.95) compared with nursing officers (2.55 ± 0.98) (*p* = 0.004). Similarly, participants who did not prefer night shift work reported significantly higher sleep disturbance scores (3.24 ± 1.03) than those who preferred night shifts (2.69 ± 0.90) (*p* = 0.001).

In Table 9, significant differences in gastrointestinal disturbances and eating habit disruptions were observed based on the highest level of education and holding a second job. Participants with a diploma reported higher mean scores (2.47 ± 0.95) compared with those holding a bachelor’s degree (2.11 ± 0.83) (*p* = 0.034). Participants without a second job also reported higher mean scores (2.43 ± 0.93) than those with a second job (2.04 ± 0.83) (*p* = 0.007). Lastly, as shown in Table 10, significant differences in cardiovascular and physical strain were observed based on preference for night shift work and holding a second job. Participants who did not prefer night shift work reported higher mean scores (2.58 ± 0.94) compared with those who preferred night shifts (2.13 ± 0.85) (*p* = 0.002). Similarly, participants without a second job reported higher scores (2.39 ± 0.90) than those with a second job (2.10 ± 0.89) (*p* = 0.040).

**Table 8:** Post-hoc comparisons of sleep disturbances scores across sociodemographic and work-related characteristics (*N* = 175).

Variable	Group Comparison	Mean ± SD	Mean difference (95% CI)	S.E. for difference	*p*-value
Religion (*p* = 0.006*)	African Traditional	1.58 ± 0.71	Ref	—	—
	Christianity	3.03 ± 0.96	1.45 (0.12-2.77)	0.56	0.028*
	Islam	2.68 ± 0.95	1.09 (-0.23-2.43)	0.56	0.130
Rank (*p* = 0.002*)	Nursing officer	2.55 ± 0.98	Ref	—	—
	Principal nursing officer	2.17 ± 0.95	-0.38 (-0.84-1.60)	0.44	0.912
	Senior nursing officer	3.27 ± 0.96	0.71 (-0.06-1.48)	0.28	0.086
	Senior staff nurse	3.24 ± 0.95	0.68 (0.15-1.20)	0.19	0.004*
	Staff nurse	2.71 ± 0.88	0.15 (-0.36-0.67)	0.18	0.919
Likes night shift work (*p* = 0.001*)	Yes	2.69 ± 0.90	Ref	—	—
	No	3.24 ± 1.03	0.55 (0.24-0.85)	0.15	0.001*

*Statistically significant at *p* < 0.05.

**Table 9:** Post-hoc comparisons of gastrointestinal disturbances and eating habit disruptions scores across sociodemographic and work-related characteristics (*N* = 175).

Variable	Group comparison	Mean ± SD	Mean difference (95% CI)	S.E. for difference	*p*-value
Highest level of nursing education (*p* = 0.034)*	Bachelor’s degree	2.11 ± 0.83	Ref	—	—
	Master’s degree	2.51 ± 1.32	0.40 (-0.57-1.37)	0.41	0.599
	Diploma	2.47 ± 0.95	0.36 (0.03-0.68)	0.13	0.029*
Currently hold a second job (*p* = 0.007)*	Yes	2.04 ± 0.83	Ref	—	—
	No	2.43 ± 0.93	0.38 (0.10-0.66)	0.14	0.007*

*Statistically significant at *p* < 0.05.

**Table 10:** Post-hoc comparisons of cardiovascular and physical strain scores across sociodemographic and work-related characteristics (*N* = 175).

Variable	Group comparison	Mean ± SD	Mean difference (95% CI)	S.E. for difference	*p*-value
Likes night shift work (*p* = 0.002)*	Yes	2.13 ± 0.85	Ref	—	—
	No	2.58 ± 0.94	0.45 (0.16-0.73)	0.14	0.002*
Currently hold a second job (*p* = 0.040)*	Yes	2.10 ± 0.89	Ref	—	—
	No	2.39 ± 0.90	0.29 (0.01-0.57)	0.13	0.039*

*Statistically significant at *p* < 0.05.

## Discussion

The aim of this research was to assess the effect of night shift work on daytime sleepiness and physiological health among pediatric nurses in the Tamale Metropolis, Northern Ghana. This study is relevant given the increasing reliance on shift work in healthcare systems and the growing evidence linking night shift duties to adverse sleep and health outcomes among nurses. Pediatric nurses, in particular, are required to maintain high levels of vigilance and clinical judgment, making adequate sleep and physiological well-being critical to patient safety and quality of care.

The study revealed that 20.6% of participants exhibited severe excessive daytime sleepiness. This implies that approximately one in five pediatric nurses in the study population may be functioning with compromised alertness during daytime hours, potentially affecting cognitive performance, decision making, and overall productivity. This finding was similarly reported in a previous study by Zabin et al.^27^, who found that 37.7% of participants experienced excessive daytime sleepiness. Another study by Haile et al.^11^ reported that 28.6% of participants also had excessive daytime sleepiness. Although the prevalence in the current study appears slightly lower, the findings consistently demonstrate that excessive daytime sleepiness is a common occupational health concern among shift-working nurses. These findings are suggestive of the persistent circadian rhythm disruption and sleep debt associated with night shift work. Therefore, there is a need for institutional strategies aimed at reducing sleep deprivation and promoting adequate rest among nurses working night shifts.

The study also revealed a moderate level of sleep disturbances associated with night shift work. While this does not indicate extreme impairment across all participants, it clearly reflects that night duties substantially interfere with normal sleep patterns. In practical terms, the disruption to sleep is still evident and functionally relevant within the study population. This finding is in agreement with past studies by Habybabady et al.^28^ and Mohamud et al.^29^, which similarly reported moderate sleep disturbances among night shift nurses. Even though the level is not severe, it remains important because persistent sleep disruption has been linked to reduced immune function, impaired concentration, increased risk of clinical errors, and long-term health consequences. Overall, these results highlight the need for interventions such as structured sleep hygiene education, improved shift rotation systems, and adequate rest intervals between shifts.

Participants also reported a low level of gastrointestinal disturbances and eating habit disruptions associated with night shift work. In spite of the low classification, the findings still suggest that night duty exerts a noticeable influence on digestive processes and dietary behaviours. This implies that even relatively minor circadian misalignment can affect meal timing, food choices, and gastrointestinal comfort. These effects may be attributed to irregular eating schedules, increased reliance on fast foods or caffeinated beverages during night shifts, and the disruption of normal circadian regulation of digestion. These findings are in line with studies by de Rijk et al.^30^ and Navruz-Varlı and Mortaş^31^, which highlighted gastrointestinal disturbances and poor dietary patterns among shift workers. Even at a low level, these changes are relevant because repeated exposure may gradually increase susceptibility to metabolic and other chronic health conditions. Therefore, ensuring access to healthy food options during night shifts and providing nutritional education remain important preventive strategies.

The study further revealed a low level of cardiovascular and physical strain associated with night shift work. Despite being categorized as low, the results indicate that night duty still imposes a measurable physiological burden on both cardiovascular and musculoskeletal systems. This suggests that the impact, while not pronounced, is nonetheless present and may accumulate with prolonged exposure. This finding is consistent with literature demonstrating that cardiovascular and physical strain are associated with night shift work^17,32^. Prolonged exposure to such strain may increase the risk of hypertension, fatigue-related musculoskeletal problems, and long-term cardiovascular complications. To address these concerns, healthcare institutions should implement targeted occupational health interventions. These may include periodic cardiovascular screening for nurses working night shifts, ergonomic assessments to reduce musculoskeletal strain, scheduled rest breaks during shifts, and workload redistribution to prevent excessive physical demands.

Significant differences in cardiovascular and physical strain were identified based on preference for night shift work. Participants who did not prefer night shift work reported higher mean cardiovascular and physical strain scores. Their non-preference might be due to pre-existing health concerns, difficulty adapting to nocturnal schedules, or heightened stress responses to night duties. Increased cardiovascular and physical strain among this group may further reinforce their negative perception of night shifts, creating a cycle of stress and physiological burden. In order to encourage adaptation and mitigate adverse outcomes, healthcare institutions should consider flexible scheduling, periodic health screening, stress management programs, and supportive workplace policies aimed at reducing the physiological burden of night shift work.

### Limitations of the study

This study has several limitations that should be considered when interpreting the findings. First, the cross-sectional design limits the ability to establish causal relationships between night shift work and daytime sleepiness or physiological health outcomes. Second, data were collected using self-reported questionnaires, which may be subject to recall bias and social desirability bias. Third, the study was conducted among pediatric nurses in selected health facilities within the Tamale Metropolis, which may limit the generalizability of the findings to nurses in other specialties or geographical settings. The sample was also predominantly male (68.6%), which is atypical of nursing populations. However, this distribution reflects the staffing composition within the selected facilities, where male nurses more frequently undertake night shift duties. This context should be considered when interpreting and generalizing the findings. Additionally, objective measures of sleep and physiological outcomes (e.g., clinical or biometric assessments) were not included, which could have provided more robust evidence. Unmeasured confounding variables, such as workload intensity, staffing levels, and individual coping strategies, may also have influenced the observed outcomes.

## Conclusion

Night shift work among pediatric nurses in the Tamale Metropolis is associated with increased daytime sleepiness and physiological health disturbances, with sleep disturbances emerging as the most pronounced outcome. Variations in these outcomes were observed across demographic and work-related factors, particularly among nurses who did not prefer night shifts. Overall, while night shift work remains essential for ensuring continuous pediatric care, it poses notable risks to nurses’ sleep and physiological well-being. These findings underscore the need for targeted occupational health strategies and improved shift scheduling practices to mitigate adverse effects and support the well-being of pediatric nurses.

### Recommendations

The findings of this study have important implications for hospital management, occupational health practice, and workforce planning. Healthcare institutions should review and optimize night shift scheduling practices by limiting consecutive night duties, ensuring adequate recovery time between shifts, and incorporating nurses’ shift preferences where operationally feasible. Given that nurses who did not prefer night shifts reported poorer outcomes, flexible and participatory scheduling approaches may help reduce sleep disturbances and physical strain. Routine occupational health monitoring should be strengthened for nurses engaged in night shift work. Periodic screening for sleep problems, fatigue, blood pressure changes, and early indicators of cardiovascular strain can facilitate timely intervention and prevent long-term complications. In addition, fatigue management and sleep hygiene education programs should be implemented to equip nurses with practical strategies to improve daytime sleep quality and manage excessive sleepiness. Providing structured rest breaks and designated quiet spaces during night shifts may further enhance recovery and alertness. Nutritional support initiatives are also necessary to address gastrointestinal and dietary disruptions. Hospitals should improve access to healthy meal options during night shifts and provide guidance on balanced eating patterns to reduce reliance on irregular meals, junk food, and excessive caffeine intake. Lastly, broader staff wellness programs, including stress management interventions, ergonomic improvements to reduce physical strain, and supportive supervision, should be integrated into institutional policies. Such measures will not only protect nurses’ health and well-being but also enhance patient safety, productivity, and the overall quality of pediatric healthcare services.

## Supplementary Information

Below is the link to the electronic supplementary material.

Supplementary Material 1

Supplementary Material 2

Supplementary Material 3

## Data Availability

The data that support the findings of this study are available from the corresponding author upon reasonable request.

## References

[1] Alqahtani, J. S. et al. The effect of cumulative night shift duties on insomnia, fatigue, and mental health in intensive care unit. *Heliyon* **10**, e31066. 10.1016/j.heliyon.2024.e31066 (2024). 38784539 10.1016/j.heliyon.2024.e31066 PMC11112310

[2] Alfonsi, V. et al. Sleep-Related Problems in Night Shift Nurses: Towards an Individualized Interventional Practice. *Front. Hum. Neurosci.* **15**, 644570. 10.3389/fnhum.2021.644570 (2021). 33796014 10.3389/fnhum.2021.644570 PMC8007770

[3] Dires, T. et al. Assessment of night-shift effects on nurses’ health and work performance at South Gondar zone public hospitals, 2022. *Int. J. Afr. Nurs. Sci.* **18**, 100530. 10.1016/j.ijans.2023.100530 (2023).

[4] Young, T. B. Epidemiology of daytime sleepiness: definitions, symptomatology, and prevalence. *J. Clin. Psychiatry*. **65** (Suppl 16), 12–16 (2004). 15575799

[5] Singareddy, R., Bixler, E. O. & Vgontzas, A. N. Fatigue or daytime sleepiness? *J. Clin. Sleep. Med.* **6**, 405 (2010). 20726292 PMC2919674

[6] Slater, G. & Steier, J. Excessive daytime sleepiness in sleep disorders. *J. Thorac. Dis.* **4**, 608–616. 10.3978/j.issn.2072-1439.2012.10.07 (2012). 23205286 10.3978/j.issn.2072-1439.2012.10.07 PMC3506799

[7] Chukwunonso-Ogbu, A., Fazli, S. A., Kalungi, G. & Malomo, O. Sleep deprivation and fatigue in healthcare staff: a clinical audit on the risk to patient safety. *Cureus* **17**, e96543. 10.7759/cureus.96543 (2025). 41250785 10.7759/cureus.96543 PMC12619980

[8] Grosser, L., Knayfati, S., Yates, C., Dorrian, J. & Banks, S. Cortisol and shiftwork: a scoping review. *Sleep Med. Rev.* **64**, 101581. 10.1016/j.smrv.2021.101581 (2022). 35872400 10.1016/j.smrv.2021.101581

[9] Alreshidi, S. M. & Rayani, A. M. The correlation between night shift work schedules, sleep quality, and depression symptoms. *Neuropsychiatr. Dis. Treat.* **19**, 1565–1571. 10.2147/NDT.S421092 (2023). 37440839 10.2147/NDT.S421092 PMC10335288

[10] Kaplan, A., Ozdemir, C. & Bulbul, E. Nurses’ level of sleepiness during night shift. *Int. Nurs. Rev.* **71**, 1062–1071. 10.1111/inr.12963 (2024). 38650476 10.1111/inr.12963 PMC11600514

[11] Haile, K. K., Asnakew, S., Waja, T. & Kerbih, H. B. Shift work sleep disorders and associated factors among nurses at federal government hospitals in Ethiopia: a cross-sectional study. *BMJ Open.* **9**, e029802. 10.1136/bmjopen-2019-029802 (2019). 31462478 10.1136/bmjopen-2019-029802 PMC6720246

[12] Al-Hrinat, J., Al-Ansi, A. M., Hendi, A., Adwan, G. & Hazaimeh, M. The impact of night shift stress and sleep disturbance on nurses quality of life: case in Palestine Red Crescent and Al-Ahli Hospital. *BMC Nurs.* **23**, 24. 10.1186/s12912-023-01673-3 (2024). 38185660 10.1186/s12912-023-01673-3 PMC10773077

[13] Stavås, J. A., Nilsen, K. B. & Matre, D. The association between proportion of night shifts and musculoskeletal pain and headaches in nurses: a cross-sectional study. *BMC Musculoskelet. Disord*. **25**, 67. 10.1186/s12891-024-07196-5 (2024). 38229099 10.1186/s12891-024-07196-5 PMC10790533

[14] Alreshidi, S. M. & Rayani, A. M. The correlation between night shift work schedules, sleep quality, and depression symptoms. *NDT* **19**, 1565–1571. 10.2147/NDT.S421092 (2023). 10.2147/NDT.S421092 PMC10335288 37440839

[15] Lin, S-C., Yeh, W-C., Liu, Z-X., Hsu, H-F. & Chen, J-Y. Night-shift work and its association with metabolic syndrome. *Med. (Baltim).* **104**, e43598. 10.1097/MD.0000000000043598 (2025). 10.1097/MD.0000000000043598 PMC12324026 40760617

[16] Jankowiak, S. et al. Night shift work and cardiovascular diseases among employees in Germany: five-year follow-up of the Gutenberg Health Study. *Scand. J. Work Environ. Health*. **50**, 142–151. 10.5271/sjweh.4139 (2024). 38258536 10.5271/sjweh.4139 PMC11006091

[17] Erdem, J. S. et al. Night shift work and indicators of cardiovascular risk: a systematic review and meta-analysis. *Environ. Res.* **276**, 121503. 10.1016/j.envres.2025.121503 (2025). 40164421 10.1016/j.envres.2025.121503

[18] Dartey, A. F., Tackie, V., Lotse, C. W., Lily, D. & Sagbo, F. M. Experiences of nurses and midwives with indecorously structured duty rosters at selected health facilities in Ho, Volta Region of Ghana: a qualitative study. *SAGE Open. Nurs.* **10**, 23779608241275323. 10.1177/23779608241275323 (2024). 39185503 10.1177/23779608241275323 PMC11342322

[19] Ashwaq, A. A. S. & Kholoud, S. A. A. The role of pediatric nurses in promoting child health and well-being. *J. Int. Crisis Risk Communication Res.* **2024**, 164–170. 10.63278/jicrcr.vi.634 (2024).

[20] Hosny Elshater, M., Abd -Elhamed Zaki, N., Abd-Elhamed, G. & Khamies Mohamed, A. Challenges facing pediatric nurses throughout the provision of nursing care for children with COVID-19. *Egypt. J. Health Care*. **12**, 1795–1805. 10.21608/ejhc.2021.276919 (2021).

[21] Yamane, T. *Statistics An Introductory Analysis* (Harper and Row, 1967).

[22] Johns, M. W. A new method for measuring daytime sleepiness: the Epworth sleepiness scale. *Sleep* **14**, 540–545 (1991). 1798888 10.1093/sleep/14.6.540

[23] Johns, M. W. The Epworth Sleepiness Scale. About the ESS (2025). https://epworthsleepinessscale.com/about-the-ess/.

[24] Silva, I. & Costa, D. Consequences of shift work and night work: a literature review. *Healthc. (Basel)*. **11**, 1410. 10.3390/healthcare11101410 (2023). 10.3390/healthcare11101410 PMC10218650 37239693

[25] Sukor, A. N. A. et al. A systematic review of literature on the association among sleep, cortisol level and cardiovascular health within the healthcare shift worker population. *Biomedicines* **2025**, 13. 10.3390/biomedicines13102539 (2025). 10.3390/biomedicines13102539 PMC12561395 41153819

[26] Hanif, A. et al. Shifting rhythms: a systematic review exploring the multifaceted effects of shift work and circadian disruption on employee cardiovascular health. *Cureus* **16**, e71003. 10.7759/cureus.71003 (2024). 39507145 10.7759/cureus.71003 PMC11539914

[27] Zabin, L. M. et al. Job stress and its impact on daytime sleepiness among nurses in Palestine: a cross-sectional study. *Sleep. Sci. Pract.* **9**, 28. 10.1186/s41606-025-00139-6 (2025).

[28] Habybabady, R. H., Okati-Aliabad, H. & Mohammadi, M. Prevalence of poor sleep quality and associated factors among nurses in Southeast Iran: a cross‐sectional study. *Health Sci. Rep.* **8**, e71542. 10.1002/hsr2.71542 (2025). 41332919 10.1002/hsr2.71542 PMC12665504

[29] Mohamud, R. Y. H. et al. Prevalence and associated factors of poor sleep quality among nurses in a tertiary care hospital: a cross-sectional study. *RMHP* **18**, 975–986. 10.2147/RMHP.S511543 (2025). 10.2147/RMHP.S511543 PMC11952070 40161898

[30] de Rijk, M. G. et al. The association between eating frequency with alertness and gastrointestinal complaints in nurses during the night shift. *J. Sleep Res.* **30**, e13306. 10.1111/jsr.13306 (2021). 33622018 10.1111/jsr.13306 PMC8518800

[31] Navruz-Varlı, S. & Mortaş, H. Shift work, shifted diets: an observational follow-up study on diet quality and sustainability among healthcare workers on night shifts. *Nutrients* **2024**, 16. 10.3390/nu16152404 (2024). 10.3390/nu16152404 PMC11313754 39125285

[32] Xi, J. et al. Association between night shift work and cardiovascular disease: a systematic review and dose-response meta-analysis. *Front. Public. Health*. **13**, 1668848. 10.3389/fpubh.2025.1668848 (2025). 41069800 10.3389/fpubh.2025.1668848 PMC12506678

