# Supplementary material for: The effect of night shift work on daytime sleepiness and physiological health among pediatric nurses in Northern Ghana: a cross-sectional survey
Source: Sci Rep. 2026 May 11;16:21569. doi: 10.1038/s41598-026-52977-8 (PMC13350758; doi:10.1038/s41598-026-52977-8)
Supplement: Supplementary file 1 — Supplementary Material 1 [file 41598_2026_52977_MOESM1_ESM.pdf]

QUESTIONNAIRE

Shift work and night shifts are very common in healthcare organizations worldwide. However, pediatric nurses doing shift work and night shifts are exposed to several stressors with psychological, social, physical and sleeping consequences.

We are researchers conducting this study to assess the effect of night shift work on daytime sleepiness and physiological health among pediatric nurses working in the Tamale Metropolis.

Taking part in this study is voluntary. However, your consent and response will enable us achieve our aim of identifying the impact of night shift schedules on the health and well-being of pediatric nurses.

Informed Consent

I hereby agree that to participate in the research study as a respondent and I understand that the data given by me will be kept confidential and be used only for research purpose.

Signature of respondent…………………… Date…………………….

Interviewer's signature…………………….

Please, the questionnaire comprises of five (4) sections: Sections A, B, and C. Kindly respond to all the questions. Please tick (√) the space that corresponds to your answer.

Thank you.

Part A: Sociodemographic Information

1. Age in years: ………………..
2. Gender: Male [ ] Female [ ]
3. Marital status: Single [ ] Married [ ] Divorced [ ] Separated [ ]
4. Highest level of education: Diploma [ ] Bachelor's degree [ ] Masters [ ] PhD [ ] Other specify………………………..
5. Number of children……………………………..
6. Religion: Islam [ ] Christian [ ] Other specify ……………………………
7. Work experience: <5 years [ ] 5-10 years [ ] Above 10 years [ ]
8. Rank: Staff nurse [ ] Senior staff nurse [ ] Nursing officer [ ] Senior nursing officer [ ] Principal nursing officer [ ]
9. Do you like night shift? Yes [ ] No [ ]
10. How many weeks of night shifts have you worked in the past year? 1-5 weeks [ ] 6-10 weeks [ ] 11-20 weeks [ ] More than 20 weeks [ ] I cannot remember [ ]
11. Do you currently hold a second job? Yes [ ] No [ ]

12. How many hours do you get to sleep after a night shift? Less than 4 hours [ ] 4 to 6 hours [ ] Above 6 hours [ ]

SECTION B: DAYTIME SLEEPINESS

How likely are you to doze off or fall asleep in the following situations, in contrast to feeling just tired?

This refers to your usual way of life in recent times.

Even if you haven't done some of these things recently try to work out how they would have affected you.

Use the following scale to choose the **most appropriate number** for each situation:

0 = would **never** doze

1 = **slight chance** of dozing

2 = **moderate chance** of dozing

3 = **high chance** of dozing

It is important that you answer each question as best you can.

**Situation**	**Chance of Dozing (0-3)**			
	**0**	**1**	**2**	**3**
Sitting and reading				
Watching television (TV)				
Sitting inactive in a public place				
Sitting for an hour as a passenger in a car				
Lying down in the afternoon to rest				
Sitting and talking to another person				
Sitting quietly after a lunch (no alcohol at lunch)				
Sitting in a car, stopped for a few minutes due to traffic				

SECTION C: EFFECT OF NIGHT SHIFT WORK ON PHYSIOLOGICAL HEALTH

The following questions are conditions that night shift schedule can lead you into. Which of the following did you experience as a result of running for night shift schedules?

Please answer all of the items as honestly and quickly as you can.

1 – *Never* 2 – *Rarely* 3 - *Sometimes*

4 – *Often* 5 – *Always*

**Statement**	**1**	**2**	**3**	**4**	**5**
**Subscale 1: Sleep disturbances**					
1. I sleep fewer hours after working at night.					
2. I find it hard to maintain a consistent sleep schedule while working nights.					
3. I experience poor quality sleep after night shifts.					
4. I take longer to fall asleep on days after working night duty.					
5. I wake up feeling unrested after sleeping post night shift.					
6. I struggle to stay asleep during the day after a night shift.					
7. I experience frequent sleep interruptions after night shifts.					
8. I feel drowsy or fall asleep unintentionally during the day after night duty.					
**Subscale 2: Gastrointestinal disturbances and eating habit disruptions**					
1. I experience indigestion or acid reflux after night shifts.					
2. I have irregular meal times while working night shifts.					
3. I eat at inappropriate times (e.g., midnight snacking) during night shifts.					
4. I skip meals while on night duty.					
5. I feel bloated or gassy after meals during night shifts.					
6. I eat more junk food or sugary snacks during night shifts.					
7. I drink more caffeinated beverages to stay awake at night.					
**Subscale 3: Cardiovascular and physical strain**					
1. I feel an increased heart rate while working night duty.					
2. I experience dizziness or light headedness while working nights.					
3. I feel unusually cold or hot during night shifts.					
4. I feel physically weak or shaky after working a night shift.					

5. I notice increased blood pressure or palpitations during night shifts.					
6. I feel fatigued throughout the day after night duty.					
7. I find it difficult to physically recover between night shifts.					

Thank you for your time and cooperation
