# Supplementary material for: The effect of night shift work on daytime sleepiness and physiological health among pediatric nurses in Northern Ghana: a cross-sectional survey
Source: Sci Rep. 2026 May 11;16:21569. doi: 10.1038/s41598-026-52977-8 (PMC13350758; doi:10.1038/s41598-026-52977-8)
Supplement: Supplementary file 2 — Supplementary Material 2 [file 41598_2026_52977_MOESM2_ESM.pdf]

Standardized Parameter Estimates

Means/Intercepts	Estimate	Std Error	Wald Z	Prob>|Z|
Constant → 2. I sleep fewer hours after working at night.	2.7021497	0.1630216	16.575411	<.0001*
Constant → 3. I find it hard to maintain a consistent sleep schedule while working nights.	2.3771909	0.1478516	16.078219	<.0001*
Constant → 4. I experience poor quality sleep after night shifts.	2.4903518	0.1530812	16.268172	<.0001*
Constant → 5. I take longer to fall asleep on days after working night duty.	2.5558519	0.1561353	16.369471	<.0001*
Constant → 7. I wake up feeling unrested after sleeping post night shift.	2.2476322	0.1419442	15.834623	<.0001*
Constant → 8. I struggle to stay asleep during the day after a night shift.	2.0854891	0.1346876	15.483895	<.0001*
Constant → 9. I experience frequent sleep interruptions after night shifts.	2.3446892	0.1463613	16.019875	<.0001*
Constant → 10. I feel drowsy or fall asleep unintentionally during the day after night duty.	2.3331949	0.1458355	15.998812	<.0001*
Constant → 15. Physiological effects [12. I experience indigestion or acid reflux after night shifts.]	1.8730336	0.1254507	14.930439	<.0001*
Constant → 15. Physiological effects [13. I have irregular meal times while working night shifts.]	1.8935448	0.1263274	14.989188	<.0001*
Constant → 15. Physiological effects [16. I eat at inappropriate times (e.g., midnight snacking) during night shifts.]	2.2318888	0.1412326	15.802928	<.0001*
Constant → 15. Physiological effects [17. I skip meals while on night duty.]	1.9772054	0.1299378	15.216553	<.0001*
Constant → 15. Physiological effects [18. I feel bloated or gassy after meals during night shifts.]	1.8467035	0.1243304	14.853198	<.0001*
Constant → 15. Physiological effects [19. I eat more junk food or sugary snacks during night shifts.]	2.014249	0.1315533	15.311271	<.0001*
Constant → 15. Physiological effects [20. I drink more caffeinated beverages to stay awake at night.]	1.7518777	0.1203456	14.557052	<.0001*
Constant → 15. Physiological effects [22. I feel an increased heart rate while working night duty.]	1.8397037	0.1240335	14.832311	<.0001*
Constant → 15. Physiological effects [25. I experience dizziness or lightheadedness while working nights.]	2.1095545	0.1357542	15.539517	<.0001*
Constant → 15. Physiological effects [26. I feel unusually cold or hot during night shifts.]	1.9027435	0.1267217	15.01514	<.0001*
Constant → 15. Physiological effects [27. I feel physically weak or shaky after working a night shift.]	2.0077838	0.1312707	15.294992	<.0001*
Constant → 15. Physiological effects [28. I notice increased blood pressure or palpitations during night shifts.]	1.7784131	0.1214526	14.642863	<.0001*
Constant → 15. Physiological effects [29. I feel fatigued throughout the day after night duty.]	2.1696665	0.1384346	15.672858	<.0001*
Constant → 15. Physiological effects [30. I find it difficult to physically recover between night shifts.]	2.0782562	0.1343678	15.466919	<.0001*

Loadings	Estimate	Std Error	Wald Z	Prob>|Z|
Sleep disturbances → 2. I sleep fewer hours after working at night.	0.8032146	0.0308139	26.06667	<.0001*
Sleep disturbances → 3. I find it hard to maintain a consistent sleep schedule while working nights.	0.8349787	0.0270278	30.893357	<.0001*
Sleep disturbances → 4. I experience poor quality sleep after night shifts.	0.8642939	0.023034	37.522583	<.0001*
Sleep disturbances → 5. I take longer to fall asleep on days after working night duty.	0.778086	0.0333286	23.345903	<.0001*
Sleep disturbances → 7. I wake up feeling unrested after sleeping post night shift.	0.7924832	0.0315988	25.079563	<.0001*
Sleep disturbances → 8. I struggle to stay asleep during the day after a night shift.	0.7536548	0.0363904	20.710278	<.0001*
Sleep disturbances → 9. I experience frequent sleep interruptions after night shifts.	0.7736418	0.0338866	22.830326	<.0001*
Sleep disturbances → 10.I feel drowsy or fall asleep unintentionally during the day after night duty.	0.6726091	0.0443846	15.154115	<.0001*
GIT disturbance → 15. Physiological effects [12.I experience indigestion or acid reflux after night shifts.]	0.6666754	0.0476013	14.005413	<.0001*
GIT disturbance → 15. Physiological effects [13.I have irregular meal times while working night shifts.]	0.726978	0.0416581	17.451047	<.0001*
GIT disturbance → 15. Physiological effects [16.I eat at inappropriate times (e.g., midnight snacking) during night shifts.]	0.7523931	0.0390132	19.28559	<.0001*
GIT disturbance → 15. Physiological effects [17.I skip meals while on night duty.]	0.6932118	0.0453322	15.291828	<.0001*
GIT disturbance → 15. Physiological effects [18.I feel bloated or gassy after meals during night shifts.]	0.7387453	0.0409745	18.029385	<.0001*
GIT disturbance → 15. Physiological effects [19.I eat more junk food or sugary snacks during night shifts.]	0.7399521	0.0404528	18.291739	<.0001*
GIT disturbance → 15. Physiological effects [20.I drink more caffeinated beverages to stay awake at night.]	0.7641355	0.0374537	20.40211	<.0001*
Cardiovascular and physical strain → 15. Physiological effects [22.I feel an increased heart rate while working night duty.]	0.6516142	0.0488111	13.349718	<.0001*
Cardiovascular and physical strain → 15. Physiological effects [25.I experience dizziness or lightheadedness while working nights.]	0.6923374	0.0441978	15.664539	<.0001*
Cardiovascular and physical strain → 15. Physiological effects [26.I feel unusually cold or hot during night shifts.]	0.7716825	0.0361926	21.321533	<.0001*
Cardiovascular and physical strain → 15. Physiological effects [27.I feel physically weak or shaky after working a night shift.]	0.8313422	0.029144	28.525291	<.0001*
Cardiovascular and physical strain → 15. Physiological effects [28.I notice increased blood pressure or palpitations during night shifts.]	0.7834187	0.0343522	22.80547	<.0001*
Cardiovascular and physical strain → 15. Physiological effects [29.I feel fatigued throughout the day after night duty.]	0.7082508	0.0431682	16.406772	<.0001*
Cardiovascular and physical strain → 15. Physiological effects [30.I find it difficult to physically recover between night shifts.]	0.7878703	0.0347308	22.685059	<.0001*

Variances	Estimate	Std Error	Wald Z	Prob>|Z|
2. I sleep fewer hours after working at night. ↔ 2.I sleep fewer hours after working at night.	0.3548463	0.0495003	7.1685734	<.0001*
3. I find it hard to maintain a consistent sleep schedule while working nights. ↔ 3.I find it hard to maintain a consistent sleep schedule while working nights.	0.3028106	0.0451352	6.708964	<.0001*
4. I experience poor quality sleep after night shifts. ↔ 4.I experience poor quality sleep after night shifts.	0.252996	0.0398162	6.3540908	<.0001*
5. I take longer to fall asleep on days after working night duty. ↔ 5.I take longer to fall asleep on days after working night duty.	0.3945821	0.051865	7.6078656	<.0001*
7. I wake up feeling unrested after sleeping post night shift. ↔ 7.I wake up feeling unrested after sleeping post night shift.	0.3719704	0.050083	7.4270816	<.0001*
8. I struggle to stay asleep during the day after a night shift. ↔ 8.I struggle to stay asleep during the day after a night shift.	0.4320044	0.0548516	7.8758817	<.0001*
9. I experience frequent sleep interruptions after night shifts. ↔ 9.I experience frequent sleep interruptions after night shifts.	0.4014784	0.0524322	7.6571037	<.0001*
10. I feel drowsy or fall asleep unintentionally during the day after night duty. ↔ 10.I feel drowsy or fall asleep unintentionally during the day after night duty.	0.547597	0.059707	9.1714112	<.0001*
15. Physiological effects [12. I experience indigestion or acid reflux after night shifts.] ↔ 15. Physiological effects [12.I experience indigestion or acid reflux after night shifts.]	0.5555439	0.0634692	8.7529711	<.0001*
15. Physiological effects [13. I have irregular meal times while working night shifts.] ↔ 15. Physiological effects [13.I have irregular meal times while working night shifts.]	0.4715031	0.0605691	7.7845499	<.0001*
15. Physiological effects [16. I eat at inappropriate times (e.g., midnight snacking) during night shifts.] ↔ 15. Physiological effects [16.I eat at inappropriate times (e.g., midnight snacking) during night shifts.]	0.4339047	0.0587066	7.3910769	<.0001*
15. Physiological effects [17. I skip meals while on night duty.] ↔ 15. Physiological effects [17.I skip meals while on night duty.]	0.5194574	0.0628496	8.2650886	<.0001*
15. Physiological effects [18. I feel bloated or gassy after meals during night shifts.] ↔ 15. Physiological effects [18.I feel bloated or gassy after meals during night shifts.]	0.4542554	0.0605395	7.5034613	<.0001*
15. Physiological effects [19. I eat more junk food or sugary snacks during night shifts.] ↔ 15. Physiological effects [19.I eat more junk food or sugary snacks during night shifts.]	0.452471	0.0598663	7.5580287	<.0001*
15. Physiological effects [20. I drink more caffeinated beverages to stay awake at night.] ↔ 15. Physiological effects [20.I drink more caffeinated beverages to stay awake at night.]	0.416097	0.0572395	7.2694057	<.0001*
15. Physiological effects [22. I feel an increased heart rate while working night duty.] ↔ 15. Physiological effects [22.I feel an increased heart rate while working night duty.]	0.5753989	0.063612	9.0454482	<.0001*
15. Physiological effects [25. I experience dizziness or lightheadedness while working nights.] ↔ 15. Physiological effects [25.I experience dizziness or lightheadedness while working nights.]	0.5206689	0.0611995	8.5077288	<.0001*
15. Physiological effects [26. I feel unusually cold or hot during night shifts.] ↔ 15. Physiological effects [26.I feel unusually cold or hot during night shifts.]	0.4045061	0.0558585	7.2416271	<.0001*
15. Physiological effects [27. I feel physically weak or shaky after working a night shift.] ↔ 15. Physiological effects [27.I feel physically weak or shaky after working a night shift.]	0.3088702	0.0484573	6.3740654	<.0001*
15. Physiological effects [28. I notice increased blood pressure or palpitations during night shifts.] ↔ 15. Physiological effects [28.I notice increased blood pressure or palpitations during night shifts.]	0.3862552	0.0538244	7.1762158	<.0001*
15. Physiological effects [29. I feel fatigued throughout the day after night duty.] ↔ 15. Physiological effects [29.I feel fatigued throughout the day after night duty.]	0.4983808	0.0611478	8.150425	<.0001*
15. Physiological effects [30. I find it difficult to physically recover between night shifts.] ↔ 15. Physiological effects [30.I find it difficult to physically recover between night shifts.]	0.3792603	0.0547267	6.9300735	<.0001*
Sleep disturbances ↔ Sleep disturbances	1	0	.	.
GIT disturbance ↔ GIT disturbance	1	0	.	.
Cardiovascular and physical strain ↔ Cardiovascular and physical strain	1	0	.	.

Assess Measurement Model

Composite Reliability

Latent Variable	Omega
Sleep disturbances	0.9279
GIT disturbance	0.8866
Cardiovascular and physical strain	0.8989

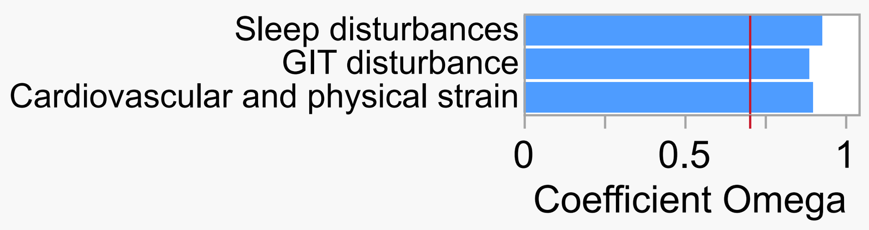

Construct Maximal Reliability

Latent Variable	H
Sleep disturbances	0.9330
GIT disturbance	0.8885
Cardiovascular and physical strain	0.9057

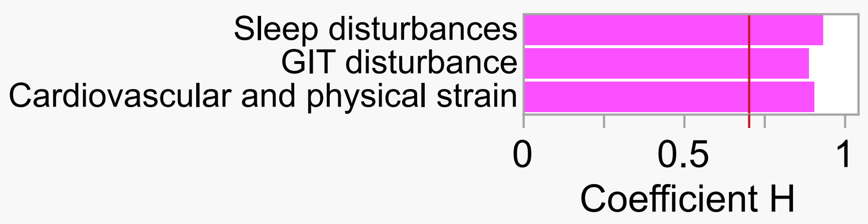

Construct Validity Matrix

	Sleep disturbances	GIT disturbance	Cardiovascular and physical strain
Sleep disturbances	0.6177	0.0000	0.0000
GIT disturbance	0.0000	0.5281	0.0000
Cardiovascular and physical strain	0.0000	0.0000	0.5610

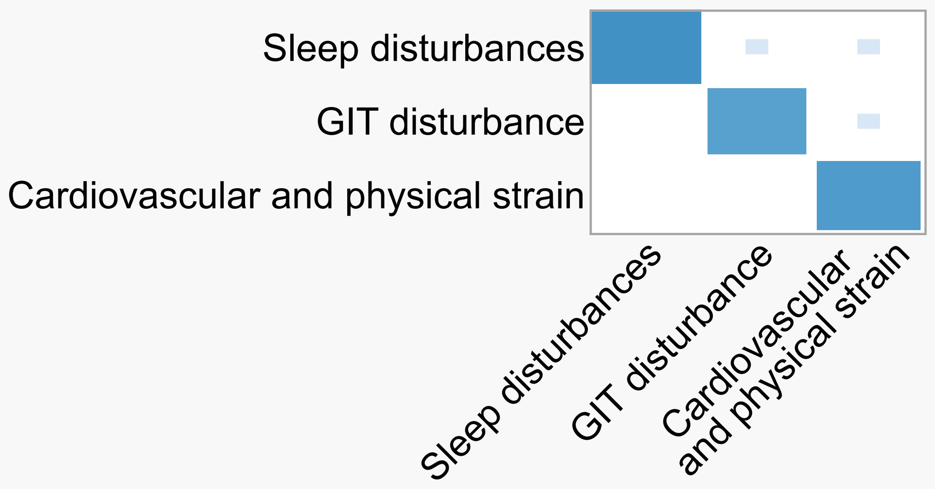

Latent variable (LV) correlations are below the diagonal, average variance extracted by the LVs are in the diagonal, and squared factor correlations of LVs are above the diagonal.
