# Supplementary material for: The effect of night shift work on daytime sleepiness and physiological health among pediatric nurses in Northern Ghana: a cross-sectional survey
Source: Sci Rep. 2026 May 11;16:21569. doi: 10.1038/s41598-026-52977-8 (PMC13350758; doi:10.1038/s41598-026-52977-8)
Supplement: Supplementary file 3 — Supplementary Material 3 [file 41598_2026_52977_MOESM3_ESM.pdf]

Term	Estimate	Std Error	t Ratio	Prob>|t|	Lower 95%	Upper 95%	VIF
Intercept	8.747644	3.538334	2.47	0.0145*	1.757707	15.73758	.
AGE[>30]	0.639968	1.082236	0.59	0.5552	-1.497975	2.777911	1.79481
GENDER[female]	-1.296304	1.039104	-1.25	0.2141	-3.349041	0.756433	1.43199
3. Marital status[married]	0.648073	1.072637	0.6	0.5466	-1.470909	2.767054	1.72150
4. Highest level of nursing education[Master's degree]	3.472462	3.361373	1.03	0.3032	-3.167891	10.11281	5.80306
4. Highest level of nursing education[Bachelor's degree]	-0.531544	1.117171	-0.48	0.6349	-2.738501	1.675413	4.32364
5. Number of children[1]	0.135251	1.78847	0.08	0.9398	-3.39785	3.668352	1.76989
5. Number of children[2]	0.012265	1.90961	0.01	0.9949	-3.760146	3.784676	1.44267
5. Number of children[3]	-1.906586	2.183592	-0.87	0.3839	-6.220247	2.407075	1.63010
6. Religion[African Traditional]	2.472516	4.070724	0.61	0.5445	-5.569151	10.51418	4.59353
6. Religion[Islam]	-2.074752	0.879255	-2.36	0.0195*	-3.81171	-0.337795	3.07643
7. Work experience[< 5 years]	-1.373065	2.619875	-0.52	0.601	-6.548597	3.802466	2.65471
7. Work experience[5-10 years]	0.10514	2.381736	0.04	0.9648	-4.59995	4.81023	1.56740
8. Rank[Nursing officer]	-1.758527	1.504075	-1.17	0.2441	-4.729809	1.212756	4.62859
8. Rank[Principal nursing officer]	-3.280828	3.738813	-0.88	0.3816	-10.66681	4.105152	3.47061
8. Rank[Senior nursing officer]	0.899582	2.106029	0.43	0.6699	-3.260853	5.060017	4.85085
8. Rank[Senior staff nurse]	-0.383771	1.112868	-0.34	0.7307	-2.582229	1.814687	5.11511
9. Do you like night shift?[No]	2.334067	0.972464	2.4	0.0176*	0.412976	4.255158	1.27815
11. Do you currently have a second job (besides this nursing role)?[No]	1.818399	0.920357	1.98	0.0500*	0.000244	3.636554	1.21702

12. On average, how many hours of sleep do you get after a night shift?[4-6 hours]	1.722882	1.487342	1.16	0.2485	-1.215344	4.661108	1.14193
12. On average, how many hours of sleep do you get after a night shift?[less than 4 hours]	1.749968	1.490363	1.17	0.2421	-1.194227	4.694162	1.22257
